# The regulation of mechanosensory motile cilium formation

**DOI:** 10.1186/2046-2530-4-S1-O7

**Published:** 2015-07-13

**Authors:** A Jarman, P zur Lage, G Mali, P Mill

**Affiliations:** 1 Centre for Integrative Physiology, University of Edinburgh, Edinburgh, UK; 2 MRC Human Genetics Unit, Institute of Genetics and Molecular Medicine, University of Edinburgh, Edinburgh, UK

## 

In contrast to the progress in understanding ciliogenesis and cilium function, we know less about the pathways for generating ciliary diversity. *Drosophila *has a variety of sensory neurons with ciliary dendrites that are structurally and functionally specialised for receiving different sensory modalities. For instance, chordotonal (Ch) neurons have mechanosensory ciliary dendrites and are required for proprioception and hearing. Time-course gene expression profiling of differentiating Ch neurons allowed us to characterise the roles of two transcription factors for ciliogenic gene regulation: the well-known cilia gene regulator, Rfx, and a factor of the Forkhead family (Fd3F), which appears to be a diverged homologue of FOXJ1. Fd3F and Rfx cooperate to regulate a cohort of genes required for ciliary motility - in *Drosophila *this is a specialisation unique to Ch neuron cilia and is essential for the hearing mechanism. Analysing the target genes of Fd3F has led to the implication of new factors in the assembly of axonemal dynein complexes. Two of these are also mutated in human primary ciliary dyskinesia. Further analysis of these genes in *Drosophila *and mouse suggests that *ZMYND10 *may be linked to an emerging chaperone pathway, while *HEATR2 *appears to have a distinct function related to transport.

